# Does Cannabinoid Use Reduce Opioid Utilization Among Patients with Gastrointestinal Cancer? Evidence from Epic COSMOS

**DOI:** 10.3390/cancers18071110

**Published:** 2026-03-30

**Authors:** Eshetu B. Worku, Selamawit A. Woldesenbet, Timothy M. Pawlik

**Affiliations:** Department of Surgery, The Ohio State University Wexner Medical Center and James Comprehensive Cancer Center, Columbus, OH 43210, USA; eshetu.worku@osumc.edu (E.B.W.); selamawit.woldesenbet@osumc.edu (S.A.W.)

**Keywords:** cannabis, cannabinoids, opioids, GI cancer, chemotherapy, COSMOS

## Abstract

Purpose: Whether medical cannabis reduces opioid use during early chemotherapy remains unclear. We examined cannabinoid and opioid prescribing among patients with GI cancers initiating chemotherapy. Methods: Patients with GI cancers initiating chemotherapy (2016–2025) were identified from the Epic COSMOS database. Cannabis exposure was defined as prescriptions for FDA-approved cannabinoids recorded in the EHR; non-prescription cannabis obtained through dispensaries were not captured. Patients with any cannabis or opioid use in the prior year or death within 90 days were excluded. Multivariable logistic regression evaluated factors associated with cannabinoid use. Results: Among 144,981 patients, 2.4% received cannabinoids within 90 days of chemotherapy initiation. Cannabinoid recipients were more likely to receive opioids than nonrecipients (60.6% vs. 31.1%, *p* < 0.01). In adjusted analyses, cannabinoid prescribing was more common among Black patients (aOR 1.45; 95%CI 1.30–1.61), women (aOR 1.11; 95%CI 1.03–1.19), and individuals with pancreatic cancer (aOR 3.26; 95%CI 2.97–3.58). Prescribing varied by region and declined over time, relative to 2017. Conclusions: Early cannabinoid prescribing was not associated with reduced opioid use. Use was more common among patients with pancreatic cancer and among Black patients, highlighting clinical and structural variation in supportive-care practices.

## Simple Summary

Patients with gastrointestinal (GI) cancers receiving chemotherapy often experience difficult symptoms such as pain, nausea, reduced appetite, and fatigue. As cannabis use has expanded across the United States, many have questioned whether it could help to manage these symptoms while also reducing reliance on opioid medications, which carry risks of dependence and overdose. Despite increasing interest, it remains unclear whether cannabis use leads to lower opioid use in cancer care. This study examined real-world prescribing patterns of cannabinoids and opioids in a large national database of patients starting chemotherapy. We aimed to understand which patients receive cannabis, how prescribing varies by region or patient characteristics, and whether cannabis use is associated with reduced opioid use early in treatment. Our findings show that cannabis prescribing remains relatively uncommon and was not linked to lower opioid use. These results underscore the need for additional research to guide safe, effective, and equitable symptom management for people undergoing cancer treatment.

## 1. Introduction

Over the past two decades, cannabis use in the United States has increased substantially, driven by state-level medical and recreational legalization and growing public acceptance [1,2,3,4]. As of 31 December 2025, medicinal cannabis is permitted in 47 states and Washington, DC, and recreational use is legal in 24 states [5]. Concurrently, cannabis is increasingly considered for symptom management (pain, nausea, vomiting, appetite loss, sleep disturbances and anxiety) [6,7]. Of note, federal deliberations in late 2025 regarding potential rescheduling from Schedule I to Schedule III further reflect the evolving recognition of possible medical utility [8]. Cannabis has also gained traction in public and clinical discourse as a potential adjunct or alternative to opioids for symptom control [9,10].

Despite these trends, opioid-related harm remains substantial [11,12]. In 2022, approximately 110,900 opioid overdose deaths were reported nationally, with synthetic opioids (e.g., fentanyl) accounting for the majority [8,9]. Whether cannabis meaningfully reduces opioid utilization, or opioid-related harms, among medically complex populations such as patients undergoing chemotherapy remains an unresolved question at the intersection of clinical care, public health policy, and drug regulation. This uncertainty underscores the need to evaluate the broader implications of medical cannabis for clinical practice, public health, and policy and regulation.

Evidence on the medical benefits of cannabis is mixed. Randomized trials and observational studies generally demonstrate modest, often clinically marginal effects across many indications [13,14,15]. Meta-analyses related to chronic pain and sleep have noted small, short-term improvements, with higher rates of nonserious adverse events such as dizziness and somnolence [15]. One Cochrane review reported that there was no consistent superiority of THC-dominant, CBD-dominant, or balanced formulations over a placebo for neuropathic pain [14]. Evidence for an opioid-sparing effect is also limited, with randomized trials demonstrating little benefit and observational estimates being inconsistent with low certainty [16]. In oncology, cannabinoids demonstrate a modest benefit for refractory chemotherapy-induced nausea and vomiting, although tolerability constrains routine use; evidence is strongest (yet heterogeneous) for spasticity in multiple sclerosis [4,17,18].

Patients with GI cancers undergoing chemotherapy often require multimodal treatment for pain, nausea, and fatigue [19]. Despite increasing cannabis availability, the prevalence of cannabis use in this population and its association with opioid utilization after chemotherapy initiation remain poorly characterized. We therefore characterized patterns of cannabis prescribing patterns at chemotherapy initiation and its association with short-term opioid utilization among patients with GI cancers, using multisite electronic health record (EHR) data.

## 2. Methods

### 2.1. Study Design, Study Population, and Data Sources

Patients with GI (colon, biliary, liver, pancreas, and rectum) cancer diagnosed between 1 January 2016 and 30 September 2025 were identified from the Epic COSMOS database [20]. COSMOS is a nationwide, multisite electronic health record (EHR) research network that aggregates de-identified patient-level data from participating Epic health systems across the United States, capturing longitudinal demographics, diagnoses, medications, procedures, and geographic characteristics to support real-world evidence generation [20]. GI cancers were defined using the International Classification of Diseases (ICD-9/10) codes and include esophagus, stomach, small intestine, colon, rectum, liver, gallbladder, biliary tract, and pancreas [21,22].

The index date was the date of chemotherapy initiation. To capture new exposure, patients with any documented cannabis (cannabinoid) or opioid prescriptions from 1 year to 15 days before the index date were excluded. Patients who died within 90 days of the index date or had incomplete medication records during follow-up were also excluded. The Ohio State University Institutional Review Board approved the study and waived informed consent because COSMOS provides de-identified, limited datasets for research.

### 2.2. Exposure

“Cannabis use” was defined as receipt of an FDA-approved cannabinoid (dronabinol, nabilone, or cannabidiol) documented on EHR medication lists or prescription fills totaling >2 days within 90 days after the index date. Exposure was binary (yes/no). Cannabis obtained via dispensaries or recreational markets is not captured in COSMOS and was therefore not included in the exposure definition.

### 2.3. Covariates

Patient-level factors included age, sex, race/ethnicity (White, Black, Hispanic, or Other [i.e., Asian, American Indian, and Alaska Native]), cancer type, U.S. Census region (Midwest, Northeast, South, West), rural versus metropolitan residence, marital status, and index year. Clinical factors included GI cancer site, treatment phase (active chemotherapy vs. postoperative), and Charlson Comorbidity Index (CCI), categorized as low (≤2) or high (>2) [23]. The social vulnerability index (SVI), a county-level composite measure of socioeconomic vulnerability linked by patient residence, was categorized as low, medium, or high, based on national tertiles [24]. The cannabis and opioid measures were derived from EHR medication lists and prescription fills within prespecified windows.

### 2.4. Outcome of Interest and Measures

The primary outcome was receipt of any opioid prescription during the same 90-day post-chemotherapy period. Among opioid recipients, cumulative opioid exposure was quantified as the total number of prescription days supplied during the follow-up period. Secondary outcomes included (1) opioid discontinuation (cessation of opioid prescriptions within 90 days after cannabis initiation) and (2) ≥50% reduction in the mean daily dose compared with the baseline. Temporal trends in cannabis prescribing were examined by calendar year, and geographic variation was described at the state level (Supplementary Table S2). Counts <11 were censored per COSMOS reporting.

### 2.5. Statistical Analysis

Continuous variables are reported as medians with interquartile ranges (IQRs), and categorical variables are reported as frequencies and percentages. Baseline differences between cannabinoid recipients and nonrecipients were assessed using the Kruskal–Wallis test for continuous variables and chi-square or Fisher’s exact tests for categorical variables. Within-person pre–post comparisons were evaluated using paired *t* tests or Wilcoxon signed-rank tests.

Multivariable logistic regression examined factors associated with early cannabis prescribing. Model 1 adjusted for patient-level and clinical covariates (age, sex, race/ethnicity, CCI, cancer type, index year). Model 2 additionally adjusted for region. Adjusted odds ratios (aORs) and 95% confidence intervals (CIs) were reported. Temporal trends were evaluated using index-year indicators (reference = 2017).

## 3. Results

### 3.1. Baseline Characteristics of Patients in the Cohort

Among 144,981 adults with a GI cancer, colon: *n* = 49,285, 34.0%; rectum: *n* = 35,094, 24.2%; pancreas: *n* = 34,280, 23.6%; liver: *n* = 23,643, 16.3%; and biliary: *n* = 2679, 1.8%. Most individuals were male (*n* = 83,534, 57.6%) and White (*n* = 100,262, 78.7%); the median age was 69 years (IQR, 60–77). The majority resided in metropolitan areas (*n* = 115,033, 80.6%), with the largest proportions living in the South (*n* = 53,960; 37.8%) and Midwest (*n* = 35,406, 24.8%). Within 90 days of chemotherapy initiation, 3390 patients (2.4%) received an FDA-approved cannabinoid prescription of >2 days; 141,591 (97.6%) individuals did not. During the same period, 46,141 (31.8%) patients received ≥1 opioid prescriptions (Table 1).

### 3.2. Opioid Utilization by Cannabis Exposure

Early prescribed cannabinoid exposure was not associated with reduced opioid use. Compared with nonrecipients, cannabinoid recipients were more likely to receive opioids (60.6% vs. 31.1%; *p* < 0.001). Among patients who received opioids, cannabinoid recipients had longer total opioid prescription days (median 35 [IQR, 8–76] vs. 15 [IQR, 4–61] days; *p* < 0.001). Compared with nonrecipients, cannabinoid recipients were more often Black (17.2% vs. 11.9%) and less likely to be White (75.1% vs. 78.8%), more likely to reside in the South (48.9% vs. 37.5%), more likely to have pancreatic cancer (49.1% vs. 23.0%), and less likely to have colon cancer (23.3% vs. 34.3%) (all *p* < 0.001). Residence in high-SVI counties was also modestly more common among recipients, compared with nonrecipients (34.6% vs. 32.3%; *p* = 0.01) (Table 1).

### 3.3. Factors Associated with Early Cannabis Prescribing

**Figure 1:**
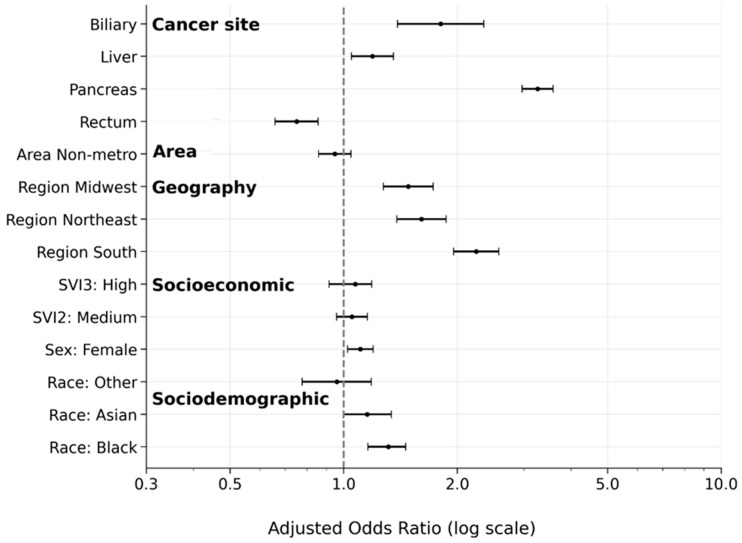
Factors associated with cannabis prescribing within 90 days of chemotherapy initiation. Horizontal forest plot with log scale and aOR = 1 reference line, point estimates with 95%CIs.

In a multivariable analysis (Model 1), Black race was independently associated with higher odds of early cannabis prescribing in a model adjusted for patient-level characteristics (Model 1: aOR 1.45, 95%CI 1.30–1.61; *p* < 0.001), an association that persisted with additional regional adjustment (Model 2, including region: aOR 1.31, 95%CI 1.18–1.46; *p* < 0.001) (Table 2 and Table 3). Female sex was also modestly associated with higher odds of cannabis use (aOR 1.11, 95%CI 1.03–1.19; *p* = 0.018). High SVI was associated with greater cannabis use in Model 1 (aOR 1.16, 95%CI 1.05–1.28; *p* = 0.009), but the effect was attenuated after adjusting for region in Model 2 (aOR 1.07, 95%CI 0.97–1.89; *p* = 0.249). Interestingly, the cancer site was the strongest determinant of early cannabis use. Compared with colon cancer, the adjusted odds were substantially higher for pancreatic cancer (aOR, 3.23; 95%CI, 2.96–3.57), modestly higher for liver cancer (aOR, 1.19; 95%CI, 1.05–1.35), and lower for rectal cancer (aOR, 0.75; 95%CI, 0.66–0.86) (all *p* < 0.001 or *p* < 0.02) (Table 2; Figure 1).

Adjusted odds ratios (aORs) and 95%CI are from a multivariable logistic regression model, including demographic characteristics, cancer site, social vulnerability, geographic region, and area of residence (Model 2). The reference categories were male sex, White race, low Social Vulnerability Index score, metropolitan residence, West region, and colon cancer. Odds ratios greater than one indicated higher odds of cannabis prescribing.

### 3.4. Geographic and Temporal Patterns

**Figure 2:**
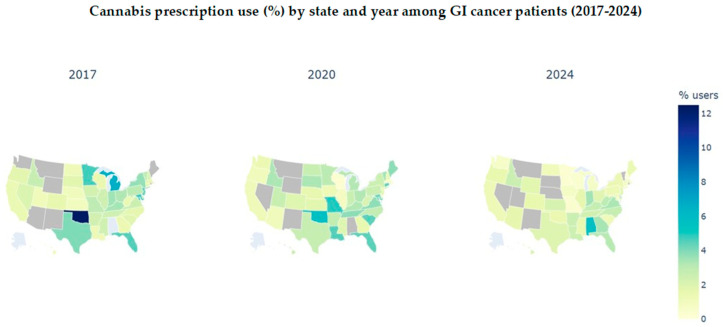
Cannabis prescription use (%) by state and year among GI cancer patients (2017–2024). The map shows overall cannabis use among GI cancer patients declining. Gray color shows numbers below 11 that are suppressed due to COSMOS regulation.

After adjustment (Model 2), substantial regional variation was observed (Table 3). Compared with the West (reference), patients in the Midwest (aOR, 1.48; 95%CI, 1.27–1.72) and Northeast (aOR, 1.61; 95%CI, 1.39–1.96) had higher odds of cannabinoid prescribing; the South also had modestly elevated odds (aOR, 1.22; *p* < 0.001; 95%CI provided in Table 3). State-level prescribing proportions varied widely (states with <11 cases were suppressed per COSMOS disclosure policies) (Supplementary Table S2; Figure 2). Relative to 2017, the odds of early cannabinoid prescribing declined over time, with lower odds by 2024 (aOR 0.66; 95%CI, 0.56–0.78) and 2025 (aOR 0.39; 95%CI, 0.32–0.48) (Table 3; Supplementary Table S3).

## 4. Discussion

Over the past two decades, the availability of medical cannabis in the United States has expanded substantially, alongside growing expectations that it may mitigate chemotherapy-related symptoms such as nausea, sleep disturbance, and appetite loss [4,25,26]. Despite extensive investigation, evidence regarding its effectiveness for cancer pain and its potential opioid-sparing role has remained mixed and inconclusive [5,12,25,26]. Notably, patient-level data evaluating prescription cannabinoids and opioid substitution during the critical period of chemotherapy initiation have been limited. Using multisite Epic COSMOS electronic health record data, the current study examined short-term opioid utilization associated with cannabinoids prescribing at chemotherapy initiation among patients with GI cancers. In this analysis, early cannabis use was not associated with reduced opioid use within 90 days; instead, THC cannabis prescribing was clustered among patients with more aggressive cancers (particularly pancreatic cancer) and varied by race and geographic region, underscoring the heterogeneity in supportive care practices and the influence of differential policy and clinical environments.

In this large, multisite EHR cohort of adults initiating chemotherapy for GI cancers, early cannabinoid prescribing was not associated with reduced short-term opioid utilization. Instead, cannabinoid recipients were more likely to receive opioids and, among opioid recipients, had a longer cumulative prescription over 90 days. These patient-level findings suggest limited short-term opioid substitution in routine GI cancer care, and align with randomized trials and meta-analyses demonstrating small or inconsistent analgesic benefits and no reliable opioid-sparing effect when cannabinoids are added to standard regimens [22,27]. Professional society guidance generally supports cannabinoids for chemotherapy-induced nausea and vomiting, with insufficient evidence for routine use in cancer-related pain or to reduce opioid exposure [28,29]. In contrast to ecological/policy-level analyses reporting declines in opioid dispensing after cannabis legalization in some populations [16,25], data in the current study indicated that such population-level associations may not reflect individual-level clinical effects during chemotherapy initiation. Collectively, these findings suggest that early cannabinoid prescribing may identify patients with greater symptom burden, rather than function as an early opioid substitute.

Cannabinoid prescribing varied by cancer site and clustered among patients with pancreatic and biliary-adjacent cancers, and it was less common in colon/rectal cancers, which is consistent with clinical triage toward patients with more severe or refractory symptoms [12,25,30]. Other observational oncology data has similarly suggested that patients with higher pain, sleep disturbance, or anxiety are more likely to seek adjunctive options, including cannabinoids, early in treatment [31,32,33,34]. Meta-analyses of randomized trials, including cancer-pain populations, have generally failed to note any meaningful incremental analgesia compared with a placebo when cannabinoids were added to opioids, alongside a higher risk of nonserious adverse events [35,36]. Within a learning health system, this pattern underscores the need for patient-level evidence and careful expectation-setting when considering cannabinoids during chemotherapy initiation.

Racial differences in prescribing persisted after adjustment, with higher odds among Black patients. These patterns mirror well-documented inequities in cancer pain management and access to supportive care [37,38,39]. Structural barriers (out-of-pocket costs, coverage exclusions, certification and travel requirements, dispensary density), clinician-level factors (implicit bias, differential risk perceptions), and patient-level determinants (stigma, prior criminalization exposure, varying beliefs about effectiveness) likely contribute to heterogeneous uptake and sourcing [18,40,41,42,43,44]. Without equity-focused implementation, legalization may yield uneven clinical and social benefits [38,45,46,47]. Oncology pathways should include coverage support, training in balanced counseling (including candid discussion of limited analgesic/opioid-sparing evidence), standardized education on safe sourcing and product labeling/potency, and social-equity provisions to mitigate disparities. Regionally, adjusted prescribing was also higher in the Midwest and Northeast, relative to the West, and the odds of cannabis prescription declined markedly after 2017. Regional heterogeneity likely reflected differences in program design and implementation (certification criteria, fees, product limits, labeling standards, dispensary density), payer coverage, and concurrent opioid policies (e.g., PDMPs, prior authorization, dose limits), rather than pharmacologic substitution alone [12,16,47]. Policy-aware oncology research should move beyond ecological inference and incorporate granular exposure measures (program features, access constraints, formulation/dose/route), patient-level outcomes, and quasi-experimental designs that account for staggered policy adoption and co-occurring regulations. Collectively, these patterns indicate that early cannabinoid prescribing is unlikely to function as an opioid-sparing strategy in routine GI cancer care and more likely reflects underlying symptom burden, underscoring the need for equitable, evidence-based supportive care interventions.

## 5. Strengths and Limitations

The current study leveraged a large, multisite EHR dataset (COSMOS), enabling the comprehensive capture of prescribing cannabinoid patterns across diverse geographic regions, racial groups, and gastrointestinal cancer types. This breadth allowed for a detailed evaluation of variation in cannabinoid use across the clinical and demographic subpopulations. In addition, robust multivariable adjustment strengthened the internal validity by addressing a wide range of potential confounders that are available within the EHR. However, despite these strengths, interpretations of the current study findings should consider several limitations. First, because COSMOS captures only FDA-approved cannabinoid prescriptions documented in the EHR, nonprescription cannabis obtained through dispensaries or state cannabis programs could not be measured, potentially leading to exposure misclassification. Second, residual confounding was likely despite exclusions and multivariable adjustment, particularly due to unmeasured symptom severity, palliative intent, and clinician prescribing preferences. Cannabinoid prescribing may function as a proxy for greater symptom burden, such as uncontrolled pain, nausea, or cachexia, rather than acting as a causal driver of opioid utilization. Third, the absence of standardized symptom-severity scores in COSMOS limited the ability to directly adjust for confounding by indication. Additionally, key contextual determinants—including state cannabis policy, insurance coverage, and product availability—were not directly linkable to patient-level records, limiting inference regarding structural drivers of use. The retrospective design precluded causal interpretation of observed associations. Regional and temporal heterogeneity further suggested evolving practice patterns within dynamic policy environments. Future studies should integrate policy and payer data, standardize cannabinoid exposure measures (dose, route, THC:CBD ratio), and examine longer-term outcomes such as opioid dose trajectories, pain interference, and functional recovery. Adoption of common data elements and learning health system approaches may accelerate evidence generation, as substantial geographic variation indicates that legalization alone is insufficient; rather, local implementation within oncology supportive care pathways likely determines the real-world impact.

## 6. Conclusions

In this large multisite EHR study of patients with GI cancers, early cannabis use at the start of chemotherapy was not associated with reduced short-term opioid utilization. Instead, cannabinoid prescribing appeared more common among patients with greater symptom burden or more aggressive disease. These patterns suggest that early cannabis use is unlikely to function as an opioid-sparing strategy in routine GI cancer care and may primarily reflect an underlying clinical need. Racial and regional differences in prescribing highlight persistent inequities and the influence of policy and practice environments, rather than true pharmacologic substitution. Overall, cannabis should be considered, when used as an adjunct for selected symptoms, rather than a replacement for opioids. Guideline-concordant multimodal analgesia, early supportive care, and equity-focused implementation remain central to effective cancer pain management.

## Figures and Tables

**Table 1:** Baseline characteristics of patients.

Characteristics	Total (*n* = 144,981)	No Cannabis Prescription (*n* = 141,591; 97.6%)	Cannabis Prescription ≥2 Days (*n* = 3390; 2.4%)	*p*-Value
Age in years	69 (60, 77)	69 (60, 77)	70 (62, 77)	**<0.001**
Sex				**<0.001**
Male	83,534 (57.6%)	81,714 (57.7%)	1820 (53.7%)	
CCI				**<0.001**
>2	141,162 (97.4%)	137,898 (97.4%)	3264 (96.3%)	
Race				**<0.001**
White	100,262 (78.7%)	98,128 (78.8%)	2134 (75.1%)	
Black	15,368 (12.1%)	14,878 (11.9%)	490 (17.2%)	
Hispanic	6160 (4.8%)	6037 (4.8%)	123 (4.3%)	
Other	5647 (4.4%)	5551 (4.5%)	96 (3.4%)	
Missing	17,544 (12.1%)	16,997 (12%)	547(16.1%)	
Region				**<0.001**
Midwest	35,406 (24.8%)	34,689 (24.9%)	717 (21.4%)	
Northeast	29,843 (20.9%)	29,181 (20.9%)	662 (19.7%)	
South	53,960 (37.8%)	52,320 (37.5%)	1640 (48.9%)	
West	23,693 (16.6%)	23,358 (16.7%)	335 (10.0%)	
Missing	2079(1.4%)	2043(1.44%)	36(1%)	
Area				**0.018**
Urban	115,033 (80.6%)	112,279 (80.6%)	2754 (82.2%)	
Rural	27,676 (19.4%)	27,080 (19.4%)	596 (17.8%)	
Missing	2272 (1.6%)	2232 (1.6%)	40 (1.2%)	
Married				**0.293**
No	57,852 (40.6%)	56,525 (40.6%)	1327 (39.7%)	
Yes	84,635 (59.4%)	82,621 (59.4%)	2014 (60.3%)	
Missing	2494(1.7%)	2445(1.7%)	49 (1.4%)	
SVI				**0.01**
Low	48,162 (33.8%)	47,096 (33.9%)	1066 (31.9%)	
Medium	48,068 (33.8%)	46,949 (33.8%)	1119 (33.5%)	
High	46,099 (32.4%)	44,942 (32.3%)	1157 (34.6%)	
Missing	2652(1.8%)	2604 (1.8%)	48(1.4%)	
Cancer site				**<0.001**
Biliary	2679 (1.8%)	2604 (1.8%)	75 (2.2%)	
Colon	49,285 (34.0%)	48,495 (34.3%)	790 (23.3%)	
Liver	23,643 (16.3%)	23,184 (16.4%)	459 (13.5%)	
Pancreas	34,280 (23.6%)	32,616 (23.0%)	1664 (49.1%)	
Rectum	35,094 (24.2%)	34,692 (24.5%)	402 (11.9%)	
Any opioid				**<0.001**
No	98,840 (68.2%)	97,506 (68.9%)	1334 (39.4%)	
Yes	46,141 (31.8%)	44,085 (31.1%)	2056 (60.6%)	
Total opioid prescription days among opioid users (Median; IQR)	16 (4–62)	15 (4–61)	35 (8–76)	**<0.001**
Total cannabis prescription days (Median; IQR)	0 (0–0)	0 (0–0)	31 (31–49)	**<0.001**
Index Year				**<0.001**
2017	11,680 (8.1%)	11,372 (8.0%)	308 (9.1%)	
2018	13,442 (9.3%)	13,069 (9.2%)	373 (11.0%)	
2019	14,058 (9.7%)	13,626 (9.6%)	432 (12.7%)	
2020	14,231 (9.8%)	13,866 (9.8%)	365 (10.8%)	
2021	16,619 (11.5%)	16,197 (11.4%)	422 (12.4%)	
2022	17,732 (12.2%)	17,301 (12.2%)	431 (12.7%)	
2023	18,284 (12.6%)	17,791 (12.6%)	493 (14.5%)	
2024	20,626 (14.2%)	20,258 (14.3%)	368 (10.9%)	
2025	18,309 (12.6%)	18,111 (12.8%)	198 (5.8%)	

Variables with >5% missingness; complete-case analyses were conducted as sensitivity analyses. Analyses were performed using SAS 9.4; two-sided α = 0.05.

**Table 2:** Multivariable model—factors associated with cannabis use (Model 1; region excluded, *p*-values adjusted for multiple comparisons).

Variable	aOR	95%CI	Adj *p*-Value
Age	1.12	0.99–1.21	0.158
Sex (male ref)			
Female	1.11	1.03–1.19	0.020
CCI (CCI ≤ 2 ref)			
CCI > 2	0.82	0.67–1.02	0.137
Race (white ref)			
Black	1.45	1.30–1.61	<0.001
Asian	0.98	0.82–1.18	0.881
Other	0.84	0.68–1.04	0.161
SVI (low ref)			
Medium	1.06	0.98–1.19	0.146
High	1.16	1.05–1.28	0.009
Area (Metro ref)			
Non-metro	0.97	0.88–1.08	0.619
Cancer site (Colon ref)			
Biliary	1.77	1.37–2.31	<0.001
Liver	1.19	1.05–1.35	0.020
Pancreas	3.23	2.96–3.57	<0.001
Rectum	0.75	0.66–0.86	<0.001
Index Year (2017 ref)			
2018	1.05	0.89–1.24	0.648
2019	1.11	0.95–1.31	0.278
2020	0.95	0.81–1.13	0.648
2021	0.91	0.77–1.07	0.326
2022	0.85	0.72–1.02	0.115
2023	1.02	0.85–1.17	0.994
2024	0.68	0.58–0.80	<0.001
2025	0.40	0.33–0.48	<0.001

Abbreviations: CI: confidence interval, Adj *p*-value: adjusted *p*-value, CCI: Charlson Comorbidity Index, and SVI: Social Vulnerability Index.

**Table 3:** Multivariable model (Model 2: Region included *p*-values adjusted for multiple comparisons).

Variable	aOR	95%CI	Adj *p*-Value
Age	1.02	0.99–1.04	0.173
Sex (Male ref)			
Female	1.11	1.03–1.19	0.018
CCI (CCI ≤ 2 ref)			
CCI > 2	0.87	0.70–1.07	0.267
Race (White ref)			
Black	1.31	1.18–1.46	<0.001
Asian	1.15	0.96–1.38	0.228
Other	0.96	0.78–1.18	0.720
SVI (SVI low ref)			
Medium	1.05	0.96–1.15	0.364
High	1.07	0.97–1.89	0.249
Region			
Midwest	1.48	1.27–1.72	<0.001
Northeast	1.61	1.39–1.96	<0.001
South	1.22	1.95–2.57	<0.001
Area (Metro ref)			
Non-metro	0.95	0.86–1.05	0.354
Cancer site (Colon ref)			
Biliary	1.83	1.39–2.35	<0.001
Liver	1.19	1.05–1.35	0.018
Pancreas	3.26	2.97–3.58	<0.001
Rectum	0.75	0.66–0.85	<0.001
Index Year (2017 ref)			
2018	1.05	0.89–1.24	0.635
2019	1.11	0.94–1.30	0.287
2020	0.95	0.81–1.23	0.635
2021	0.89	0.75–1.04	0.223
2022	0.83	0.70–0.97	0.044
2023	0.99	0.84 -1.17	0.882
2024	0.66	0.56–0.78	<0.001
2025	0.39	0.32–0.48	<0.001

Abbreviations: CI: Confidence interval, Adj *p*-value: Adjusted *p*-value, CCI: Charlson Comorbidity Index, SVI: Social Vulnerability Index.

## Data Availability

The COSMOS data used in this study are not publicly available because they are proprietary and only accessible to participating Epic health system partners. Researchers may request access to COSMOS data through Epic Systems, subject to institutional agreements and data-use approvals.

## Supplementary Materials

The following supporting information can be downloaded at: https://www.mdpi.com/article/10.3390/cancers18071110/s1, Table S1: Medications Under Investigation; Table S2: Cannabis prescriptions among GI cancer patients (2017–2024); Table S3: Year States Legalized Cannabis Policy for Recreational and/or medical programs as of 30 September 2025.
