# Supplementary material for: Does Cannabinoid Use Reduce Opioid Utilization Among Patients with Gastrointestinal Cancer? Evidence from Epic COSMOS
Source: Cancers (Basel). 2026 Mar 30;18(7):1110. doi: 10.3390/cancers18071110 (PMC13072402; doi:10.3390/cancers18071110)
Supplement: Supplementary file 1 [file cancers-18-01110-s001.zip › cancers-4190831-supplementary.pdf]

Supplementary Table S1: Medications Under Investigation

Medication	Drug Name	RxNorm Generic Name	RxCUI
Cannabis-related medications (FDA-approved cannabinoids)	Dronabinol	dronabinol	19782
	Nabilone	nabilone	7274
	Cannabidiol	cannabidiol	617321
**Opioid analgesics**	Morphine	morphine	7052
	Oxycodone	oxycodone	7804
	Hydrocodone	hydrocodone	5489
	Hydromorphone	hydromorphone	3423
	Fentanyl	fentanyl	4337
	Methadone	methadone	6813
	Codeine	codeine	2670
	Tapentadol	tapentadol	857004
	Tramadol	tramadol	10689

Supplementary Table S2: Cannabis prescriptions among GI cancer patients (2017-2024)

State	Index Year	Cannabis use (%)	N
AL	2020	*	25
AL	2024	5.68	88
AR	2017	2.35	85
AR	2,020	2.78	112
AR	2024	2.61	115
AZ	2017	*	299
AZ	2020	1.08	186
AZ	2024	1.65	242
CA	2017	1.37	876
CA	2020	1.22	1,068
CA	2024	1.31	1,683
CO	2017	1.61	186
CO	2020	1.91	200
CO	2024	1.55	322
CT	2017	1.57	382
CT	2020	4.53	375
CT	2024	0.9	430

DE	2017	2.9	69
DE	2020	2.04	49
DE	2024	3.7	54
FL	2017	4.56	439
FL	2020	4.5	578
FL	2024	3.14	1,306
GA	2017	1.41	142
GA	2020	1.52	198
GA	2024	3.38	621
HI	2017	1.05	95
HI	2020	2.68	149
HI	2024	2.15	87
IA	2017	2.29	175
IA	2020	1.25	240
IA	2024	0.78	257
ID	2017	2.67	75
ID	2020	3.33	90
ID	2024	2.58	155
IL	2017	2.45	286
IL	2020	0.88	680
IL	2024	1.66	721
IN	2017	3.51	228
IN	2020	2.65	302
IN	2024	3.49	430
KS	2017	0.97	207
KS	2020	1.81	166
KS	2024	2.19	225
KY	2017	3.42	263
KY	2020	3.24	340
KY	2024	2.75	472
LA	2017	1.3	308
LA	2020	4.55	336
LA	2024	2.52	436
MA	2017	1.77	283
MA	2020	0.63	316
MA	2024	1.11	449
MD	2017	5.1	157
MD	2020	4.39	205
MD	2024	1.33	225
ME	2017	*	35
ME	2020	3.85	52
ME	2024	1.35	74
MI	2017	6.61	257
MI	2020	3.21	655
MI	2024	0.75	802

MN	2017	4.71	255
MN	2020	2.99	301
MN	2024	0.3	329
MO	2017	2.97	232
MO	2020	4.98	201
MO	2024	0.36	278
MS	2017	2.42	207
MS	2020	1.9	290
MS	2024	1.54	456
MT	2017	*	32
MT	2020	*	37
MT	2024	*	134
NC	2017	1.7	471
NC	2020	2.82	673
NC	2024	2.7	1,407
ND	2017	1.22	82
ND	2020	2.63	126
ND	2024	1.14	88
NE	2017	1.06	94
NE	2020	1.6	125
NE	2024	*	150
NH	2017	2.08	96
NH	2020	1.46	137
NH	2024	0	125
NJ	2017	4.39	319
NJ	2020	1.65	243
NJ	2024	1.84	490
NM	2017	*	21
NM	2020	*	26
NM	2024	*	31
NV	2017	2	50
NV	2020	*	59
NV	2024	*	75
NY	2017	3.71	458
NY	2020	2.49	683
NY	2024	1.46	956
OH	2017	3.27	673
OH	2020	3.23	804
OH	2024	1.87	1123
OK	2017	12.24	49
OK	2020	5.74	122
OK	2024	1.49	268
OR	2017	1.75	171
OR	2020	1.05	191
OR	2024	1.55	258

PA	2017	3.02	1,060
PA	2020	2.59	1,005
PA	2024	1.25	1,123
RI	2017	*	89
RI	2020	0.79	127
RI	2024	*	127
SC	2017	1.83	273
SC	2020	4.51	244
SC	2024	1.22	490
SD	2017	1.79	56
SD	2020	3.33	60
SD	2024	*	52
TN	2017	2.44	164
TN	2020	3.83	183
TN	2024	2.36	339
TX	2017	4.02	722
TX	2020	2.67	938
TX	2024	1.94	1,183
UT	2017	1.14	88
UT	2020	2.63	76
UT	2024	*	123
VA	2017	2.53	356
VA	2020	3.79	572
VA	2024	3.32	534
VT	2017	2.94	34
VT	2020	3.41	88
VT	2024	*	84
WA	2017	*	119
WA	2020	1.4	143
WA	2024	0.62	162
WI	2017	1.47	270
WI	2020	0.3	330
WI	2024	0.44	454
WV	2017	1.45	138
WV	2020	2.13	141
WV	2024	2.94	204
WY	2017	*	20
WY	2020	*	22
WY	2024	1.82	55

Note: Below 11 numbers are suppressed due to cosmos regulation.

Supplementary Table S3: Year States Legalized Cannabis Policy for Recreational and/or medical programs as of 30 September 2025

State	Year Approved Recreational	Year Approved Medical	CBD-Only	THC Limit
Alabama	—	2021	No	Low-THC program (≤ 5% THC)
Alaska	2014	1998	No	
Arizona	2020	2010	No	
Arkansas	—	2016	No	
California	2016	1996	No	
Colorado	2012	2000	No	
Connecticut	2021	2012	No	
Delaware	2023	2011	No	
Florida	—	2016	No	
Georgia	—	2015	Yes	
Hawaii	—	2000	No	
Idaho	—	—	No	
Illinois	2019	2013	No	
Indiana	—	—	Yes	Low-THC CBD-only (≤ 0.3% THC hemp standard)
Iowa	—	2017	Yes	Low-THC medical program (≤ 4.5 g THC per 90 days; earlier ≤ 3% THC)
Kansas	—	—	No	
Kentucky	—	2023	Yes	Low-THC CBD-only (≤ 0.3% THC hemp standard)
Louisiana	—	2021	No	
Maine	2016	1999	No	
Maryland	2022	2014	No	

Massachusetts	2016	2012	No	
Michigan	2018	2008	No	
Minnesota	2023	2014	No	
Mississippi	—	2022	No	
Missouri	2022	2018	No	
Montana	2020	2004	No	
Nebraska	—	2024	No	
Nevada	2016	2000	No	
New Hampshire	—	2013	No	
New Jersey	2020	2010	No	
New Mexico	2021	2007	No	
New York	2021	2014	No	
North Carolina	—	—	Yes	
North Dakota	—	2016	No	Low-THC CBD-only (≤ 0.9% THC
Ohio	—	2016	No	
Oklahoma	—	2018	No	
Oregon	2014	1998	No	
Pennsylvania	—	2016	No	
Rhode Island	2022	2006	No	
South Carolina	—	—	Yes	Low-THC CBD-only (≤ 0.9% THC)
South Dakota	—	2020	No	
Tennessee	—	—	Yes	Low-THC CBD-only (≤ 0.9% THC)

Texas	—	—	Yes	Low-THC medical program (≤ 1% THC)
Utah	—	2018	No	
Vermont	2018	2004	No	
Virginia	2021	2015	No	
Washington	2012	1998	No	
West Virginia	—	2017	No	
Wisconsin	—	—	Yes	CBD-only (≤ 0.3% THC hemp standard)
Wyoming	—	—	No	

Legend: Recreational = Adult-use cannabis legal; Medical = Full medical cannabis program legal; CBD-Only = Low-THC/high-CBD only; Prohibited = No legal cannabis access
